# 2019 Novel Coronavirus Disease, Crisis, and Isolation

**DOI:** 10.3389/fpsyg.2020.01958

**Published:** 2020-08-06

**Authors:** Dev Roychowdhury

**Affiliations:** DR ACADEMY, Melbourne, VIC, Australia

**Keywords:** 2019 novel coronavirus disease, coronavirus, crisis, isolation, psychology, health, wellbeing, recommendations

## Abstract

The highly contagious 2019 novel coronavirus disease (COVID-19) outbreak has not only impacted health systems, economies, and governments, it has also rapidly grown into a global health crisis, which is now threatening the lives of millions of people globally. While, on one hand, medical institutions are critically attempting to find a cure, on the other hand, governments have introduced striking measures and policies to curtail the rapid spread of the disease. Although COVID-19 has achieved pandemic status and is predominantly viewed as a biomedical issue, it is argued that it should also be treated as a psychological crisis. This paper also reviews the literature to examine and comment on the detrimental effects of isolation, which has been enforced as one of the primary preventative measures to manage the spread of COVID-19. This paper further outlines key recommendations that should be addressed across different levels to buffer against the known adverse effects of isolation, which is especially relevant for the current COVID-19 situation, where a large proportion of the global population is isolated, confined, and/or quarantined.

## Introduction

Epidemiological research suggests that the outbreak of 2019 novel coronavirus disease, or COVID-19, has rapidly spread from a seafood and wet animal market in Wuhan (China) in December 2019 to over 216 countries worldwide in June 2020, with 7,127,753 confirmed cases and 407,159 deaths (World Health Organization, 2020a,b). The severity of the emerging COVID-19 infection and its associated zoonotic viral pathogenesis bring with them significant human costs, which includes, but are not limited to, physical, economical, mental, organizational, social, and cultural wellbeing. Although some level of psychological distress is normal and expected during times of crises, dynamic adaptation to extraordinary, prolonged, and/or uncertain levels of deleterious crisis becomes vital, especially if it causes significant disruptions and endangers global populace. This is particularly relevant now when in addition to extraordinary medical trials to find a cure for COVID-19, the world is also simultaneously witnessing a period of global crises with the enforcement of striking public health measures and policies that are likely to reorient human behavior, choices, and lifestyle for some time to come. Effective and efficient management of crises, therefore, becomes crucially important because of the impact and substantial costs to humanity when they are not resolved. The nature, scope, and impact of crises should not be limited to retrospective academic scholarship but should be proactively, systematically, and concurrently studied by the broader community.

## Crisis: Definition

The word “crisis” generally refers to a time of intense difficulty or adversity. Although a number of synonymous terms (such as catastrophe, emergency, disaster, threat, and danger) may be found in the extant literature, a scholarly definition of crisis still remains elusive. Arriving at a comprehensive understanding or consensus on a definition of crisis is not only a part of sound academic approach, but also paves the way for scientific investigation to be undertaken with the aim of explaining, predicting, and managing crisis successfully. This is especially true for COVID-19, where accurate and timely understanding of the virus, its manifestation and transmission to humans, and the crisis it engenders is of paramount importance.

WHO defines crises as a situation that is perceived as difficult, which implies the possibility of an insidious process that cannot be defined in time (World Health Organization, 2020c). Seeger et al. (1998) characterize crisis as a specific, unexpected, and nonroutine event that creates uncertainty and threat. Similarly, James and Wooten (2010) define crisis as a rare, significant, and public situation that creates highly undesirable outcomes. Although there is a consensus that crisis represents a turning point which interferes with routine business operations (Spillan and Hough, 2003), there has been some divergences in its conceptualization, where some authors argue that crisis situation can be extreme and abnormal (Pearson and Clair, 1998), while some others posit that crisis manifests as a result of a long period of incubation that bluntly occurs through the influence of a precipitating event (Roux-Dufort, 2009). For the purpose of this paper, crisis is defined as an occurrence of a solitary or series of apparent or surreptitious incident(s) or event(s) that causes an initial shock that impedes normal functioning, immediately accompanied by a state of real and/or perceived urgency, threat, and helplessness, which has the potential to cause significant disruptions and endanger individuals, communities, and/or humanity, in real-time or sometime in the near future. In proposing this definition, it is argued that crisis may eventuate as a result of one solitary blunt incident or a series of causative precipitative factors that align in a certain manner in a given time period to cause major disruptions or a combination of both with one following the other in quick succession. It is important to clarify that crisis may originate in any one domain and quickly escalate out of control and infect other domains. This is especially true for COVID-19 pandemic that emerged primarily as an epidemiological issue but has rapidly grown into a global economic and public health crisis.

While on one hand, most international bodies and institutions have ramped up measures to conduct epidemiological studies and medical trials, on the other hand, major governments and organizations have introduced new policies and legislations to curtail the spread of COVID-19 (e.g., Australian Government Department of Health, 2020; Centers for Disease Control and Prevention, 2020; World Health Organization, 2020d). The ultimate challenge in such an endeavor is to simultaneously implement a plan of action that would yield immediate and intended outcomes in the short-term while carefully balancing and managing the consequent results of such measures in the long-term. In other words, strategies and procedures must be planned, developed, and executed in a way that helps exterminate the present crisis while simultaneously averting any potential negative outcomes that may eventuate as a result of those actions in the future. Although all of the proposed guidelines and measures are intended to stop the rapid spread of COVID-19 in the short-term, the long-term health consequences of some of these measures are not known. One such measure, isolation, has been strongly recommended and legally enforced, not only just for individuals who have contracted the virus or have been in contact with a confirmed case, but also for general public who have been advised to stay indoors (Australian Medical Association, 2020; Elsworthy and Willis, 2020). Intuitively, it may appear that these measures would have the intended impact and desired outcome, but the efficacy and manner in which this would take place has been brought into question (Rodgers, 2020). This may be crucially true for isolation, which could appear to have positive short-term benefits, but also have deleterious health consequences for individuals in the long-term, especially those who have not tested positive for COVID-19 but find themselves isolated or restricted.

## Isolation and Health Consequences

Humans are social beings. And therefore, it is reasonable to argue that our need for being social is crucial for our health and wellbeing, and that any form of isolation that challenges this can be disruptive. Although the concept of isolation has received considerable attention in the literature, a comprehensive definition still remains elusive. This is further convoluted by the fact that a number of other concepts are often used interchangeably with isolation, which include loneliness, solitude, quarantine, and confinement. Walker and Avant (2011) argue that it is essential to identify distinguishing characteristics of isolation to successfully delineate it from other related concepts. So far, researchers have identified three aspects of human isolation: sensory deprivation, confinement, and social isolation (Rasmussen, 2008; Sells, 2008). Most studies maintain that these aspects of isolation, when experienced by themselves or in a combination, can cause significant decrements in adaptation and sustenance (Haythorn, 2008). Furthermore, the existing literature on isolation lacks contextual manifestation. For instance, it would be reasonable to argue that isolation due to solitary confinement in prison would be very different to isolation during space exploration or polar expedition or quarantine during a pandemic. Similarly, ontological understanding of isolation is also contingent on the subsequent epistemological viewpoint. For instance, isolation is viewed differently in the social sciences or humanities than it is in other fields of study, such as chemistry or cyber security. Providing a comprehensive coverage of how isolation has been characterized in the literature or differentiating between the various types of isolation and other related concepts is beyond the scope of this paper. However, for the purpose of this paper, a thymological view has been adopted, and isolation has been defined as a real or perceived state, where an individual experiences separation from their usual sense of being and feels limited in internal and/or external space or movement and interpersonal connections, which results in detrimental psychophysiological alterations and decrements in adaptation and performance. In light of this definition and the current circumstances that surround COVID-19 pandemic, this perspective considers the different but related concepts of loneliness, solitude, quarantine, and confinement to fall under the broader rubric of isolation.

Recent research indicates that isolation can not only be a social problem, but it can also pose serious challenges for the public health system (Klinenberg, 2016; Snell, 2017). Isolation and lack of social interaction have been consistently linked with a range of risk factors for poor health, undesirable health outcomes, increased morbidity, and early mortality (e.g., Knox and Uvnas-Moberg, 1998; Cornwell and Waite, 2009; Shankar et al., 2011; Coyle and Dugan, 2012; Luo et al., 2012; Barger, 2013; Pantell et al., 2013; Steptoe et al., 2013; Barger et al., 2014; Kreibig et al., 2014; Shevlin et al., 2014; Tsai et al., 2014; Holt-Lunstad et al., 2015; Miyawaki, 2015; Na and Hample, 2016; Valtorta et al., 2016; Chang et al., 2017; Courtin and Knapp, 2017; Rico-Uribe et al., 2018).

Similarly, numerous epidemiological, experimental, clinical, and longitudinal studies on isolation have indicated that it has profoundly detrimental effect on the psychological and physical health of individuals. Isolation has been found to cause impairment in optimal functioning in mood, cognitive performance, stress hormones, and neurological activity (Golden et al., 2009; Schneider et al., 2010; Cacioppo et al., 2015; Friedler et al., 2015). Studies examining executive functioning and working memory in socially excluded participants have reported reduced brain activity and inferior cognitive performance (Sauer et al., 1999; Reed et al., 2001; Campbell et al., 2006). Furthermore, neuroscientific studies on the effects of long-term isolation have shown that individuals may experience a range of degenerative symptoms, including neurocognitive and immune modulatory alterations, fatigue, misaligned circadian rhythm, sleep disorders, and altered stress hormone levels (e.g., Jacubowski et al., 2015; Pagel and Choukèr, 2016). Neurobiological studies have indicated that isolation may cause atrophy in certain brain areas, such as the hippocampus and the prefrontal cortex (Duman and Monteggia, 2006; Liston et al., 2009) and decrease of brain-derived neurotrophic factors (Barrientos et al., 2003; Gong et al., 2017), which are responsible for neurogenesis and plasticity. Similarly, Schneider et al. (2010) found that isolated individuals reported decreased electrocortical activity when they were subjected to long-term confinement.

Psychosociological studies have linked isolation to impaired self-regulation of hedonistic processes (Baumeister et al., 2005) and increased perception of loneliness (van Baarsen et al., 2009). It has also been found that feelings of loneliness are strong predictors for reduced cognitive performance (Tilvis et al., 2004; Wilson et al., 2007) and fragmented sleep (Cacioppo et al., 2002; Kurina et al., 2011). Numerous studies have repeatedly demonstrated that lonely individuals are more likely to worry about being evaluated negatively and feel more threatened in social situations (Cacioppo et al., 2006), experience heightened accessibility of negative social information (Cacioppo and Hawkley, 2009), and report higher sensitivity to the presence of pain (Yamada and Decety, 2009). Similarly, it has been found that loneliness is strongly associated with markers of threat surveillance (Mendes et al., 2002). This is in line with imaging studies that have linked loneliness to greater activation of visual cortex in response to negative social images (Cacioppo et al., 2009) and eye tracking research that has shown that lonely individuals are more likely to spend a greater proportion of their time fixating on socially threatening stimuli in a social scene (Bangee et al., 2014).

It is evident that isolation causes significant neurophysiological and psychosocial disruptions, which can have serious implications for the health and wellbeing of individuals. Considering the severe biopsychosocial outcomes, it would be reasonable to state that isolation has the potential to seriously and negatively influence individuals’ cognitive capabilities and decision-making and problem-solving abilities, along with deterioration in interpersonal relationships and overall quality of life. This may be particularly true for the current COVID-19 situation, which has already witnessed discrepancies in how individuals have behaved around the world – while some individuals have chosen to ignore critical health recommendations and advice (Bhanot, 2020; Pollock et al., 2020), others have indulged in stocking up on essential supplies (Boulet and Kodikara, 2020; Lufkin, 2020) – both presenting challenge, panic, and uncertainty in the society.

This present perspective seeks to encourage critical discourse analysis and practices in this domain, especially to inform research and policy. More specifically, this paper is intended to serve as a caution that in addition to the ongoing medical and economic crises, we are also in the midst of a palpable psychological crisis, which if unchecked, can further burden the already enervated state of reality we find ourselves in. In light of the proposed definition of crisis, it is argued that the current COVID-19 pandemic presents significant and unprecedented risk of a *global isolation crisis*, which will present substantial mental, social, economic, and public health challenges. It is, therefore, imperative to acknowledge and address global isolation due to COVID-19 as a (psychological) health crisis. This is particularly significant for, but is not limited to, majority of those individuals who have not tested positive for COVID-19 but have been subjected to isolation. For a large proportion of these individuals, the indefinite period of isolation coupled with the uncertainty of financial, medical, and social wellbeing can instigate real and/or perceived crisis. It should also be noted that the present perspective is not intended to criticize the lockdown measures that have been implemented globally and shown to be effective in containing the spread of the virus. Rather, it is hoped that this perspective will also highlight that the interim solution of isolation may potentially proliferate other vulnerabilities and hence warrants further investigation and analysis.

## Discussion and Recommendations

Given the deleterious effects of isolation and the serious consequences they may have on the health and wellbeing of individuals, it is argued that it must be treated as a crisis and that a collaborative, concerted, and committed effort must be made to buffer against the known adverse effects of isolation. These measures must be developed and implemented across various levels of the society to ensure that we are prepared for arduous and uncertain times. This is especially relevant for the current COVID-19 situation, where a large proportion of the global population is isolated and quarantined.

Considering the limitations found in the extant literature reviewed, this paper addresses a number of shortcomings and proposes practical and workable solutions to address isolation crisis. It is evident that the conceptualization of isolation in the literature has largely been unsystematic and unidimensional. Although previous research has identified a number of indicators of isolation, a comprehensive and multidimensional understanding of this construct still remains elusive. Moreover, researchers in this domain have primarily focused on obtaining and interpreting quantifiable aspects of isolation, such as frequency and duration of isolation, and as such have largely disregarded the qualitative contexts where isolation manifests. Similarly, the vast majority of research on isolation has been conducted on Western population, particularly the US and UK, and as such does not represent the lifestyle, socioeconomic circumstances, and cultural diversity of the global population. Also, a large proportion of the research examining health risks associated with isolation has primarily focused on the elderly population. The academic and research community should, therefore, carefully examine, characterize, and define isolation and its correlates, both at micro- and macro-levels. This also includes, but is not limited to, examining other related concepts, such as loneliness, solitude, quarantine, and confinement. Furthermore, ethnographic, phenomenological, cross-cultural, and longitudinal studies must be conducted to understand how isolation affects the psychophysiology of individuals across the lifespan, especially in different contexts, such as voluntary and non-voluntary isolation, and short-term and long-term isolation. Future research must also focus on understanding the chronology of effects during initial period of incubation along with its manifestations along different time intervals (i.e., short-term, medium- to long-term, and long-term) so that appropriate measures could be suitably developed.

Equipped with this knowledge, health professionals and counselors should develop tailored programs and interventions to assist high-risk individuals. For instance, community engagement programs may be developed to assist children in orphanages or elderly people in aged-care facilities to engage in regular physical activity and maintain adequate dietary and nutritional intake during periods of isolation and confinement, especially in times of crisis such as COVID-19. Also, a routine check-in program may be introduced at the local or community level, where allied health professionals connect with residents to ascertain their level of functioning and provide them with appropriate care and resources. This is especially relevant for the current COVID-19 pandemic, where most people now find themselves isolated for an indefinite period of time, which is likely to present psychophysiological challenges. Also, efficient use of technology (such as telehealth or ehealth sessions), social media (such as Facebook or Twitter), and collaborative platforms (such as Skype or Zoom) in this scenario may greatly alleviate logistical, health, or communication concerns, especially considering the physical distancing rule that has been implemented to curtail the spread of COVID-19. Furthermore, novel and innovative ways of encouraging social interaction and inclusivity must be developed and promoted so that individuals are not only able to optimally function and reap the social and health benefits of interpersonalism, but also converge in solidarity during times of crisis such as COVID-19.

Additionally, organizations and governments across the world must collaborate to develop multidimensional indices of isolation, which also factor the influence of other related variables, such as age, gender, multimorbidity, and lack of social integration. Also, crisis modeling systems and interventions to forecast and detect impairment due to isolation must be developed as essential precautionary measures. Furthermore, merging national and international data from previous crises along with current data, a real-time and dynamic database must be maintained that provides accurate and timely information on the state of crisis and best practices for management and mitigation. This database should also contain historical information on the physiological effects of isolation on the human body and its reaction to periods of isolation and confinement. Preparatory and educational measures and policies must be developed *apriori* so that individuals are equipped to take necessary steps to assuage the known adverse effects of isolation and crisis. This would not only help mitigate adverse effects for the individuals, but also alleviate tremendous burden that would otherwise strain the public health system during times of crisis.

Finally, in an attempt to develop preventative infection control measures and health policies, we must also acknowledge that COVID-19 affects women, men, and children differently. This pandemic is likely to worsen the existing vulnerabilities of certain groups of people who are already at a disadvantage. This may include, but is not limited to, individuals who have faced gender inequality or been subjected to domestic or intimate partner violence; marginalized groups such as people with disabilities and people of color; and those who are homeless, refugees, or in extreme poverty. This makes COVID-19 a major human rights issue where these groups of people are at a disproportionately higher probability of being adversely affected. And therefore, it is critical that measures that are intended to resolve the COVID-19 conundrum must also mindfully intersect with basic and universal human rights, especially for those who have been isolated, marginalized, alienated, or disempowered.

## Conclusion

In a span of few months, COVID-19 pandemic has emerged as a major biomedical threat to global economy and public health systems. Given its transmissibility, it is imperative that strict infection control and safety measures are adopted across the globe. Considering the timeframe required to develop effective vaccine, isolation has been asserted as a fundamental measure to limit the contagion. But prolonged, indefinite, and uncertain periods of isolation may bring with it adverse psychophysiological effects. In this paper, it is argued that emerging global psychological distress and isolation due to COVID-19 should also be treated as a crisis. Appropriate precautionary and mitigatory measures must be developed and introduced at various levels in the society, which would not only aid efforts at individual and community levels, but also help reduce burden on the already encumbered public health systems.

## Data Availability Statement

The original contributions presented in the study are included in the article/supplementary material, further inquiries can be directed to the corresponding author/s.

## Author Contributions

The author confirms being the sole contributor of this work and has approved it for publication.

### Conflict of Interest

The author declares that the research was conducted in the absence of any commercial or financial relationships that could be construed as a potential conflict of interest.
